# Prognostic value of decreased FOXP1 protein expression in various tumors: a systematic review and meta-analysis

**DOI:** 10.1038/srep30437

**Published:** 2016-07-26

**Authors:** Jian Xiao, Bixiu He, Yong Zou, Xi Chen, Xiaoxiao Lu, Mingxuan Xie, Wei Li, Shuya He, Shaojin You, Qiong Chen

**Affiliations:** 1 Department of Geriatrics, Respiratory Medicine, Xiangya Hospital of Central South University, Changsha, China; 2 Department of Respiratory Medicine, Xiangya Hospital of Central South University, Changsha, China; 3 Department of Biochemistry and Biology, University of South China, Hengyang, China; 4 Laboratory of Cancer Experimental Therapy, Atlanta Research & Educational Foundation (151F), Atlanta VA Medical Center, Decatur, GA, USA

## Abstract

The prognostic value of forkhead box protein P1 (FOXP1) protein expression in tumors remains controversial. Therefore, we conducted a systematic review and meta-analysis, searching the PubMed, Embase and Web of Science databases to identify eligible studies. In total, we analyzed 22 articles that examined 9 tumor types and included 2468 patients. Overall, decreased expression of FOXP1 protein was associated with favorable overall survival (OS) in lymphoma patients (HR = 0.38, 95%CI: 0.30–0.48, p < 0.001). In patients with solid tumors, decreased FOXP1 expression correlated with unfavorable OS (HR = 1.82, 95%CI: 1.18–2.83, p = 0.007). However, when FOXP1 protein expression was nuclear, decreased expression was also associated with favorable OS (HR = 0.53, 95%CI: 0.32–0.86, p = 0.011). Furthermore, decreased FOXP1 expression resulted in the best OS in patients with mucosa-associated lymphoid tissue (MALT) lymphomas (HR = 0.26, 95%CI: 0.11–0.59, p = 0.001), but the worst OS was observed in non-small cell lung cancer (NSCLC) patients (HR = 3.11, 95%CI: 1.87–5.17, p < 0.001). In addition, decreased FOXP1 expression was significantly correlated with an unfavorable relapse-free survival (RFS) in breast cancer patients (HR = 1.93, 95%CI: 1.33–2.80, p = 0.001).

Forkhead box protein P1 (FOXP1) is a protein encoded by the *FOXP1* gene^1^ that belongs to the forkhead box transcription factor family^2^. Functioning as a transcriptional repressor, FOXP1 regulates a program of gene repression that is essential for myocardial development^3^. In addition, FOXP1 is also a crucial regulator in the development of the lung, esophagus, cortical neuron, hair follicle and jaw tissues^4^,^5^,^6^,^7^,^8^.

Aside from a critical role in regulating the development of normal human tissues, FOXP1 is also involved in tumorigenesis. In diffuse large B-cell lymphomas (DLBCL), FOXP1 suppresses immune response signatures and promotes tumor cell survival to act as an oncoprotein^9^,^10^. However, in other types of tumors, such as neuroblastoma and prostate cancer, FOXP1 can inhibit cell growth and attenuate tumorigenicity to exert a tumor-suppressive effect^11^,^12^. Thus, the function of FOXP1 in tumor development and progression is inconsistent.

Similarly, this contradiction is also demonstrated in the prognostic value of FOXP1 protein expression in tumor patients. Decreased FOXP1 protein expression in DLBCL or mucosa-associated lymphoid tissue (MALT) lymphoma patients is associated with favorable survival^13^,^14^,^15^. However, in patients with breast, endometrial or non-small cell lung cancer (NSCLC), the decreased FOXP1 expression is correlated with poor survival^16^,^17^,^18^. Therefore, we carried out this systematic review and meta-analysis to explore the cause of these inconsistent observations and determine the prognostic value of decreased FOXP1 protein in patients with various tumors.

## Methods

This systematic review and meta-analysis was conducted according to the PRISMA statement^19^.

### Search strategy

We systematically searched in the online PubMed, Embase and Web of Science databases (updated until May 6, 2016) with the restrictions of English language and article format. The following keywords or their combinations were used in the searches: “FOXP1 OR forkhead box protein 1” AND “survival OR prognosis OR prognostic” AND “cancer OR tumor OR tumour OR neoplasm OR neoplasma OR neoplasia OR carcinoma OR cancers OR tumors OR tumours OR neoplasms OR neoplasmas OR neoplasias OR carcinomas OR leukemia OR leukemias OR leukaemia OR leukaemias OR lymphoma OR lymphomas”. Additional studies were identified by referring to relevant articles to avoid omissions due to electronic searching.

### Study selection criteria

Eligible studies in our meta-analysis were selected according to the following criteria: (1) full text original studies published in English that measured the FOXP1 protein expression in patients with tumors without restricting the type of cancer; (2) the protein expression was determined by immunohistochemistry (IHC); (3) results included the determination of a correlation between FOXP1 expression and patient survival; (4) the hazard ratios (HRs) and their corresponding 95% confidence intervals (CIs) were either reported or calculated using other information (e.g., survival curves); and (5) when repeated results were reported by the same authors, we included the most complete report. However, patient survival outcomes in this meta-analysis included overall survival (OS), cancer-specific survival (CSS), relapse-free survival (RFS), progression-free survival (PFS), disease-free survival (DFS) and failure-free survival (FFS, which was defined as in Nyman’s study^20^ that evaluated survival from the date of diagnosis until relapse or death of any cause). Additionally, unpublished studies, meeting abstracts, comments, letters, case reports, literature reviews and meta-analyses were excluded.

### Quality assessment

In correspondence to a critical review checklist that was proposed by Meta-analysis of Observational Studies in Epidemiology (MOOSE) group issued by Dutch Cochrane Centre^21^ and referencing Zhou’s study^22^, we used the following quality control criteria: (1) specific definition of study population; (2) specific description of study design; (3) sample size greater than 30; (4) specific definition of survival outcome such as OS, CSS, RFS, PFS, DFS and FFS; (5) specific definition of the cut-off value for decreased FOXP1 protein expression; and (6) sufficient follow-up time.

### Data extraction

Two investigators (Jian Xiao and Bixiu He) independently extracted the primary information according to a predefined form, which included the following sub-categories: first author, year of publication, country of study population, tumor type, sample source, test method, location of FOXP1 protein expression, cut-off value, sample size, follow-up time, survival outcome, analysis method and HR estimation. When both multivariate and univariate analyses of the OS results were performed, HRs and their corresponding 95%CIs were extracted preferentially from the multivariate analyses. If HR and its corresponding 95%CI were not directly reported, they were calculated and estimated using the previously reported methods^23^. All disagreements were discussed until a consensus was reached.

### Statistical analysis

We used STATA 12.0 software (Stata Corporation, College Station, TX, USA) to perform all of the statistical analyses. The extracted HRs and their corresponding 95%CIs were comprehensively calculated to obtain pooled HRs and 95%CIs. If the pooled HR > 1 as well as its 95%CI did not overlap with 1, the decreased expression of the FOXP1 protein would be considered as an indicator for the poor survival prognosis in tumor patients. Analysis of the heterogeneity of the combined HRs was carried out using Cochran’s Q test and Higgins’ I-squared statistic. Heterogeneity was defined as I^2^ > 50% or p < 0.05. If heterogeneity was present, a random-effects model was conducted. If not, the fixed-effects model would be applied. Sensitivity analysis was performed to assess the stability of the results. Furthermore, subgroup analysis and meta-regression were adopted to explore the sources of the heterogeneity. In addition, the publication bias was evaluated by Begg’s and Egger’s tests. However, all of the p values in our results were two-tailed, and p < 0.05 was considered to be statistically significant.

## Results

### Study selection

**Figure 1:**
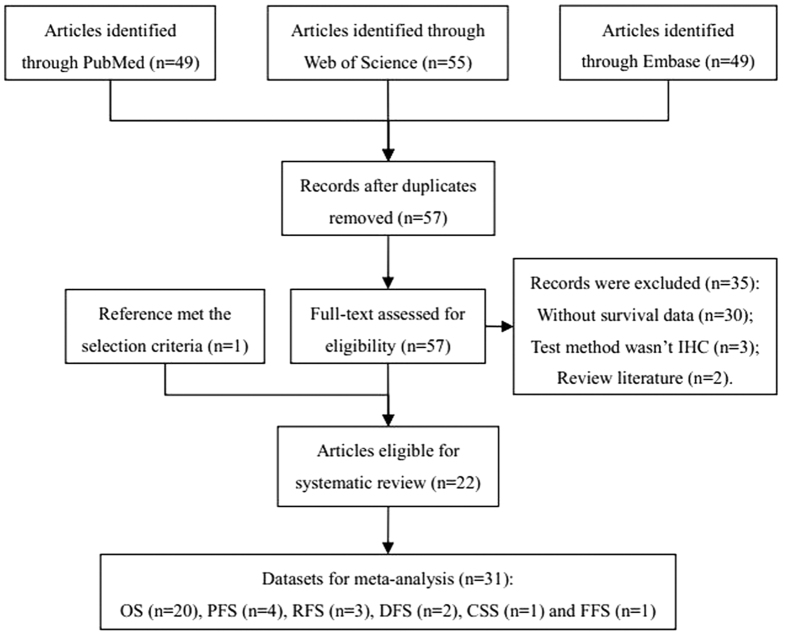
Flow diagram for study identification.

The initial database searching identified one hundred and fifty-three potentially relevant records. After the duplicates were removed, fifty-seven records remained. By assessing the full text for eligibility, thirty-five of these studies were excluded because they did not conform to the selection criteria. However, one additional study that also met our selection criteria was obtained from the references of relevant articles. Thus, a total of twenty-two studies were included in this systematic review. Finally, thirty-one datasets were used to perform the meta-analysis (Fig. 1).

### Characteristics of the included studies

The characteristics of the 22 included studies are summarized in Tables 1 and 2. In total, 2468 tumor patients from 9 different countries were included in our meta-analysis, and the studies were published from 2004 to 2015. The tumor types contained are as follows: DLBCL^13^,^14^,^15^,^20^,^24^,^25^,^26^,^27^,^28^,^29^, breast cancer^16^,^30^,^31^, endometrial cancer^17^, MALT lymphoma^14^,^32^,^33^,^34^, hepatocellular carcinoma^35^, NSCLC^18^, prostate cancer^12^, colorectal cancer^36^ and epithelial ovarian cancer^37^. As for the survival outcomes, 22 eligible studies were divided into 31 datasets: 20 for OS, 4 for PFS, 3 for RFS, 2 for DFS, 1 for CSS and 1 for FFS (Table 1 and Fig. 1). However, the cut-off value for the decreased expression of FOXP1 protein was inconsistent among these eligible studies (Table 2).

### Meta-analysis of OS

**Figure 2:**
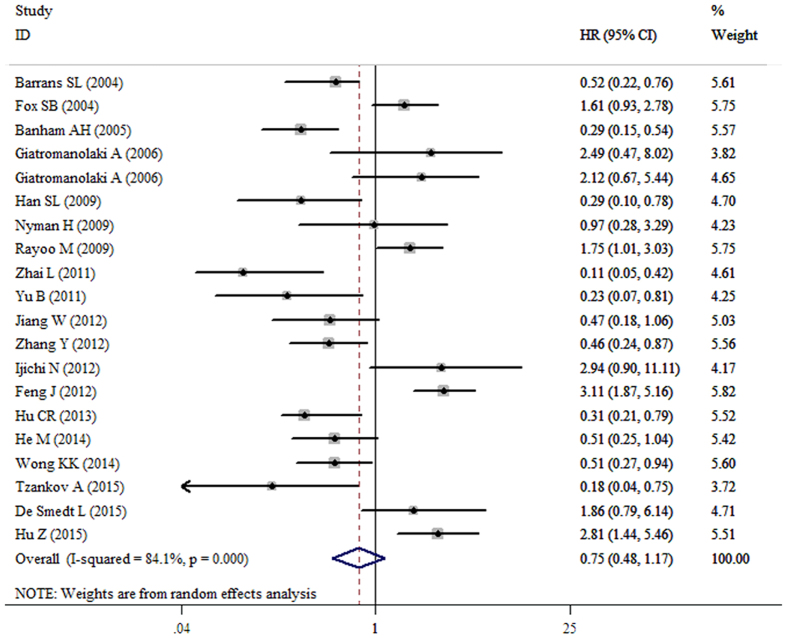
Forest plot for the relationships between decreased FOXP1 protein expression and OS in all tumor patients included in this meta-analysis.

**Figure 3:**
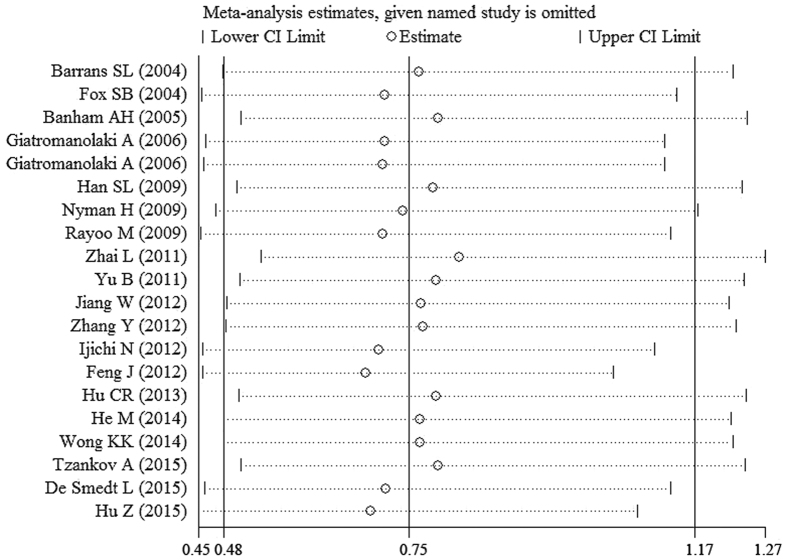
Sensitivity analysis for the meta-analysis of OS in all tumor patients included in this meta-analysis.

**Figure 4:**
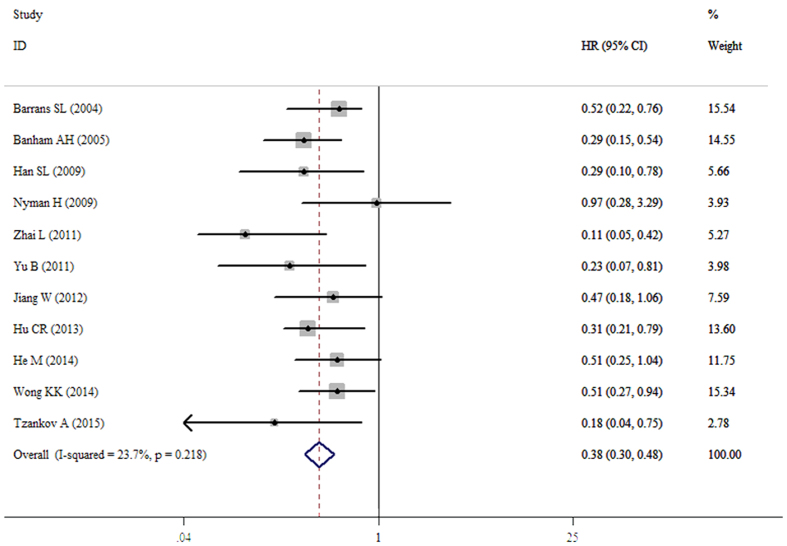
Forest plot for the relationships between decreased FOXP1 protein expression and OS in lymphoma patients.

**Figure 5:**
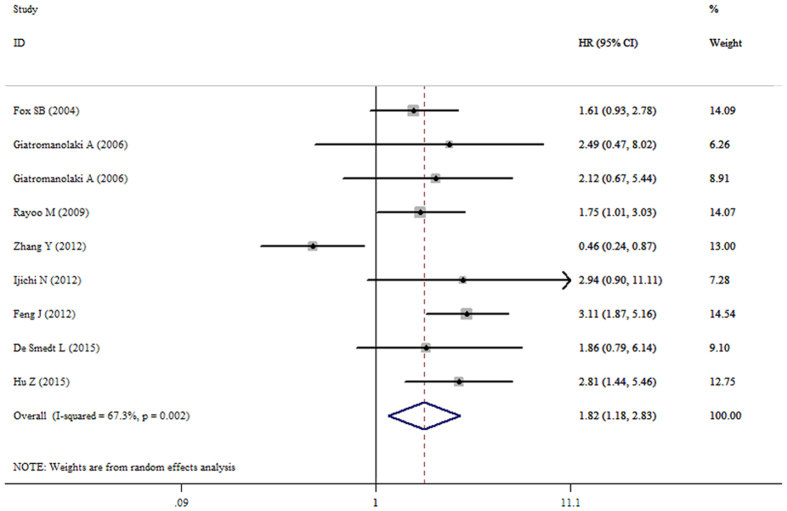
Forest plot for the relationships between decreased FOXP1 protein expression and OS in patients with solid tumors.

The pooled result from twenty datasets yielded no significant association between decreased FOXP1 protein expression and OS in patients with various tumors (HR = 0.75, 95%CI: 0.48–1.17, p = 0.203) (Table 3 and Fig. 2). A sensitivity analysis was performed by successively omitting each study, and the results revealed the pooled HRs did not vary substantially after excluding any individual study (Fig. 3), which implied that the pooled OS HR was stable. However, in the subgroup analyses based on cancer type (which included DLBCL and MALT lymphoma) and solid tumors (which excluded DLBCL and MALT lymphoma), the pooled results demonstrated that decreased FOXP1 expression had a favorable prognostic value for lymphomas (HR = 0.38, 95%CI: 0.30–0.48, p < 0.001) but an unfavorable prognosis for solid tumors (HR = 1.82, 95%CI: 1.18–2.83, p = 0.007) (Figs 4 and 5). Furthermore, when the FOXP1 protein was expressed in the nucleus, decreased FOXP1 expression indicated a good prognosis for OS (HR = 0.53, 95%CI: 0.32–0.86, p = 0.011) (Table 3).

It is interesting that decreased expression of FOXP1 had different prognostic values for lymphomas and solid tumors. To reveal this contradictory phenomenon, we further conducted subgroup analyses for both of these cancer types. As shown in Table 4 for the subgroup analyses results for lymphomas, decreased FOXP1 expression had the best OS in patients with MALT lymphoma (HR = 0.26, 95%CI: 0.11–0.59, p = 0.001). However, decreased FOXP1 protein expression in patients with solid tumors was associated with a significantly worse OS in most of the subgroup categories, and the worst OS was observed in NSCLC patients (HR = 3.11, 95%CI: 1.87–5.17, p < 0.001) (Table 5).

### Meta-analysis of CSS/DFS/FFS/PFS/RFS

**Figure 6:**
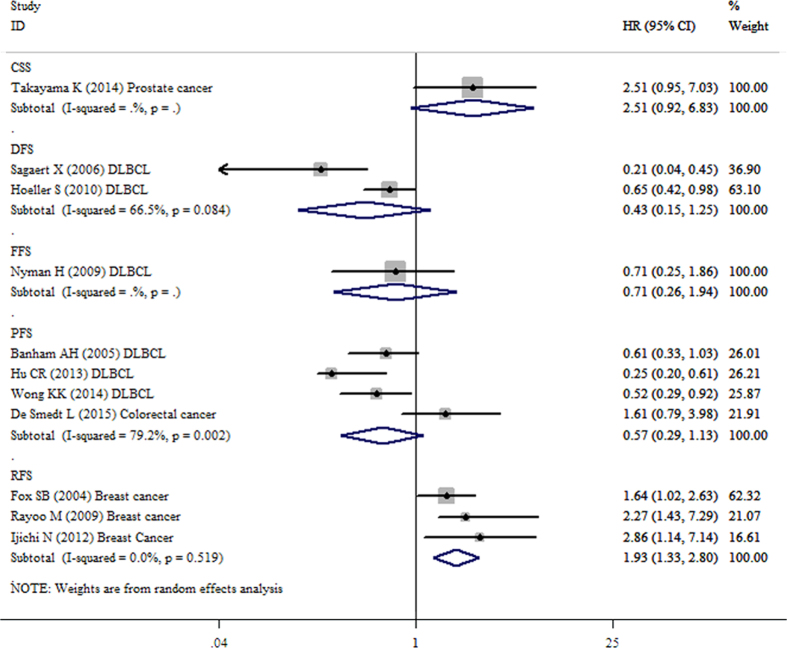
Forest plot for the relationships between decreased FOXP1 protein expression and CSS/DFS/FFS/PFS/RFS.

Both the CSS for prostate cancer and the FFS for DLBCL were derived from only one dataset and neither showed significant associations with the decreased FOXP1 protein expression (HR = 2.51, 95%CI: 0.92–6.83, p = 0.071; HR = 0.71, 95%CI: 0.26–1.94, p = 0.504, respectively). The pooled results from two datasets for the DFS for DLBCL and four datasets for the PFS for DLBCL and colorectal cancer also indicated no statistical significance (HR = 0.43, 95%CI: 0.15–1.25, p = 0.120; HR = 0.57, 95%CI: 0.29–1.13, p = 0.107, respectively). However, in patients with breast cancer, the pooled result of three datasets showed that decreased FOXP1 expression was significantly correlated with an unfavorable RFS (HR = 1.93, 95%CI: 1.33–2.80, p = 0.001) (Fig. 6).

### Meta-regression analysis of OS

To investigate the source of heterogeneity among OS datasets (I^2^ = 84.1%, p < 0.001), we performed meta-regression analyses by choosing variables such as publication year, country, cancer type, sample source, expression location, sample size and analysis method. The results suggested that cancer type (residual I^2^ = 6.26%, adjusted R^2^ = 100.00%) and expression location (residual I^2^ = 80.68%, adjusted R^2^ = 24.29%) were the major sources of significant heterogeneity among datasets regarding OS (Supplementary Table S1). Consequently, as cancer type can almost completely explain the heterogeneity among OS datasets, the subgroup analyses for it showed that the heterogeneities were much lower (Tables 3, 4, 5).

### Publication bias

**Figure 7:**
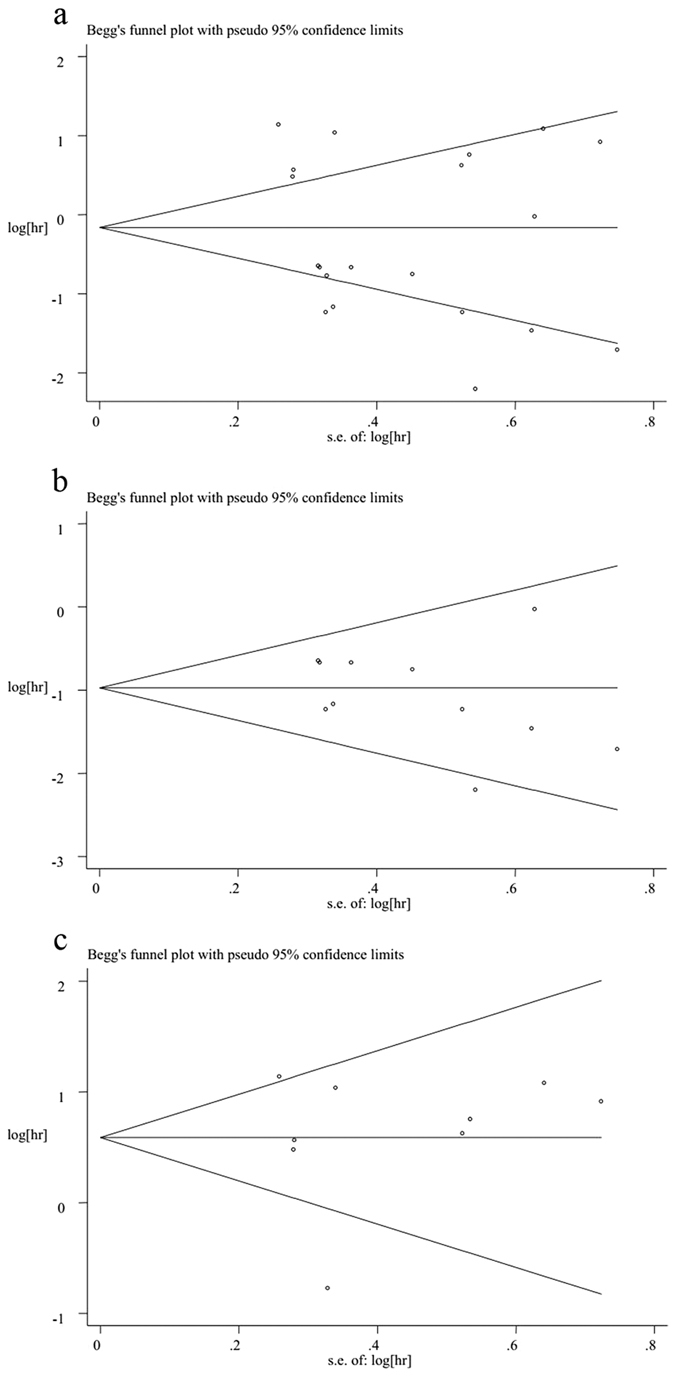
(**a**) Begg’s funnel plot of publication bias for meta-analysis of OS in all tumor patients included in this study; (**b**) Begg’s funnel plot of publication bias for meta-analysis of OS in patients with lymphomas; (**c**) Begg’s funnel plot of publication bias for meta-analysis of OS in patients with solid tumors.

As the amount of datasets for meta-analysis of CSS/DFS/FFS/PFS/RFS were fewer (each of them were less than five), we only evaluated the publication bias for the OS meta-analysis. However, both Begg’s funnel plot and Egger’s linear regression test were used to evaluate the publication bias. The results indicated that no publication bias in all of the OS datasets for all tumor types (p = 0.347 for Begg’s test and p = 0.275 for Egger’s test). Publication bias also did not exist in the datasets regarding the OS for lymphomas (p = 0.213 for Begg’s test and p = 0.291 for Egger’s test) or solid tumors (p = 0.602 for Begg’s test and p = 0.864 for Egger’s test) (Fig. 7).

## Discussion

FOXP1 plays an important role during pathologic tumor development by potentiating Wnt/β-catenin signaling in DLBCL^38^. By repressing S1PR2 signaling, FOXP1 also promotes the survival of DLBCL cells^10^. In addition, FOXP1 negatively regulates androgen receptor signaling in prostate cancer to function as an androgen-responsive transcription factor^39^. Furthermore, FOXP1 still serves as an oncogene through promoting the cancer stem cell-like characteristics of ovarian cancer cells^40^. All of these observations indicate that the FOXP1 protein may have a specific prognostic value for tumor patients. However, thus far, no consistent conclusion has been made^14^,^15^,^16^,^18^.To the best of our knowledge, this is the first meta-analysis examining the prognostic value of decreased FOXP1 protein in various tumors.

Our meta-analysis incorporated 22 eligible studies with 31 datasets. The survival data included OS, PFS, RFS, DFS, CSS and FFS. First, we found no significant association between decreased FOXP1 protein expression and OS in patients with various tumors. When the subgroup analyses were conducted, the pooled results demonstrated that decreased FOXP1 expression was a favorable prognostic factor for lymphomas but an unfavorable factor for solid tumors. However, if the FOXP1 protein expression was located in the nucleus, decreased FOXP1 expression indicated a good OS prognosis. Furthermore, the results showed that decreased FOXP1 expression was correlated with the best OS in patients with MALT lymphoma but associated with the worst OS in NSCLC patients. Additionally, in patients with solid tumors such as breast cancer, decreased FOXP1 expression was also significantly correlated with an unfavorable RFS. It should be noted that no publication bias was found in this meta-analysis.

Several important implications were confirmed by our study. First, decreased FOXP1 protein expression may be a universal favorable prognostic factor for lymphomas. In this meta-analysis, we included the lymphoma type, such as DLBCL^13^,^14^,^15^,^20^,^24^,^25^,^26^,^27^,^28^,^29^ and MALT lymphoma^14^,^32^,^33^,^34^, and the results were also confirmed by studies with chronic lymphocytic leukemia^41^. Thus, we speculate that decreased FOXP1 protein expression may have similar prognostic value for all types of lymphoma that originate from lymphocytes. Second, decreased expression of FOXP1 is an unfavorable factor for solid tumors. As the meta-analysis results were pooled from breast cancer^16^,^30^,^31^, endometrial cancer^17^, hepatocellular carcinoma^35^, NSCLC^18^, prostate cancer^12^, colorectal cancer^36^ and epithelial ovarian cancer^37^, and combined with further evidence from neuroblastoma^11^, we considered that this finding may be applicable to all solid tumors. Third, FOXP1 protein may function as a tumor promoter in lymphomas and act as a tumor suppressor in solid tumors. However, further research into these mechanisms is needed to verify this inference. Additionally, solid tumor patients with decreased FOXP1 protein expression in tumor tissues may indicate sensitivity to chemotherapy. Studies *in vitro* found that down-regulated FOXP1 expression can improve the sensitivity to chemotherapy in tumor cells^37^,^40^,^42^. Thus, we speculate that these situations may also occur in patients with solid tumors. However, more *in vivo* experiments are needed to confirm our speculation.

In this meta-analysis, we wanted to study the prognostic value of decreased FOXP1 protein expression in various tumors. However, we did not comprehensively evaluate the prognostic impact of overexpressed FOXP1 protein in the tumor patients. The major reason for this is that all of the eligible studies included in our study had defined decreased FOXP1 expression (Table 2), whereas relatively few studies^15^,^18^,^35^,^36^ had reported an association between the overexpression of FOXP1 and survival outcome in tumor patients. Therefore, to highlight the key point of decreased FOXP1 expression, we only focused on the prognostic value of decreased FOXP1 protein expression in our current meta-analysis. However, as more original studies regarding the association between the overexpression of FOXP1 and survival outcomes in tumor patients will be conducted, a systematic study on the prognostic value of overexpressed FOXP1 protein in tumor patients can also be performed in the future.

There are some limitations that should be noted in our meta-analysis. The tumor types for both lymphomas and solid tumors included in this meta-analysis are limited, and our results should be cautiously extended to other specific tumor types. We only recruited articles published in English, thus a language bias might exist. Some HRs and their corresponding 95%CIs were extracted from the survival curves. However, these data are less reliable than those directly obtained from survival data. Because of the lack of data, the meta-analysis results regarding the CSS/DFS/FFS/PFS/RFS should be updated when more related studies are completed. Finally, studies regarding various tumors without a consistent cut-off value may be restricted to expand the clinical applicability^43^,^44^,^45^,^46^. Therefore, a unified cut-off value for the decreased FOXP1 protein is warranted.

In summary, our meta-analysis suggests that decreased expression of the FOXP1 protein is associated with better survival in patients with lymphomas but poorer survival in patients with solid tumors. However, further prospective studies with larger sample sizes are required to validate the prognostic value of decreased FOXP1 expression in various tumors.

## Additional Information

**How to cite this article**: Xiao, J. *et al*. Prognostic value of decreased FOXP1 protein expression in various tumors: a systematic review and meta-analysis. *Sci. Rep*. **6**, 30437; doi: 10.1038/srep30437 (2016).

## Supplementary Material

Supplementary Information

## Figures and Tables

**Table 1:** Main characteristics for the studies included in the meta-analysis.

First author	Year	Country	Cancer type	Sample source	Test method	Expression location	Sample size	Follow-up, Median (range)	Outcome	Analysis method	HR estimation
Barrans SL^15^	2004	UK	DLBCL	FFPE	IHC	Nucleus	126	NR	OS	Univariate	SC
Fox SB^30^	2004	UK	Breast cancer	TMA	IHC	Nucleus and cytoplasm	283	87.6 (2.4–135.6)	OS	Multivariate	Reported
Fox SB^30^	2004	UK	Breast cancer	TMA	IHC	Nucleus and cytoplasm	283	87.6 (2.4–135.6)	RFS	Multivariate	Reported
Banham AH^24^	2005	Canada	DLBCL	TMA	IHC	Nucleus	109	NR	OS	Multivariate	Reported
Banham AH^24^	2005	Canada	DLBCL	TMA	IHC	Nucleus	109	NR	PFS	Univariate	SC
Sagaert X^25^	2006	Belgium	DLBCL	FFPE	IHC	Nucleus	68	NR	DFS	Univariate	SC
Giatromanolaki A^17^	2006	Greece	Endometrial cancer	FFPE	IHC	Nucleus	82	70 (22–182)	OS	Univariate	SC
Giatromanolaki A^17^	2006	Greece	Endometrial cancer	FFPE	IHC	Cytoplasm	82	70 (22–182)	OS	Univariate	SC
Nyman H^20^	2009	Finland	DLBCL	FFPE	IHC	Nucleus	117	29 (7–64)	FFS	Univariate	SC
Han SL^32^	2009	China	MALT lymphoma	FFPE	IHC	Nucleus	43	NR	OS	Univariate	SC
Nyman H^20^	2009	Finland	DLBCL	FFPE	IHC	Nucleus	117	29 (7–64)	OS	Univariate	SC
Rayoo M^16^	2009	Australia	Breast cancer	TMA	IHC	Nucleus	121	64 (NR)	OS	Multivariate	Reported
Rayoo M^16^	2009	Australia	Breast cancer	TMA	IHC	Nucleus	121	64 (NR)	RFS	Univariate	SC
Hoeller S^26^	2010	Switzerland	DLBCL	TMA	IHC	Nucleus	167	NR	DFS	Univariate	SC
Zhai L^33^	2011	China	MALT lymphoma	FFPE	IHC	Nucleus	50	68.4 (6.8–167.0)	OS	Univariate	SC
Yu B^13^	2011	China	DLBCL	FFPE	IHC	Nucleus	35	42 (2–108)	OS	Univariate	SC
Jiang W^34^	2012	China	MALT lymphoma	FFPE	IHC	Nucleus	92	NR	OS	Univariate	SC
Zhang Y^35^	2012	China	Hepatocellular carcinoma	TMA	IHC	Nucleus and cytoplasm	114	NR	OS	Multivariate	Reported
Ijichi N^31^	2012	Japan	Breast cancer	FFPE	IHC	Nucleus and cytoplasm	113	NR	OS	Multivariate	Reported
Feng J^18^	2012	China	NSCLC	TMA	IHC	Nucleus and cytoplasm	101	NR	OS	Multivariate	Reported
Ijichi N^31^	2012	Japan	Breast Cancer	FFPE	IHC	Nucleus and cytoplasm	113	NR	RFS	Multivariate	Reported
Hu CR^27^	2013	China	DLBCL	FFPE	IHC	Nucleus	92	20 (1–58)	OS	Univariate	SC
Hu CR^27^	2013	China	DLBCL	FFPE	IHC	Nucleus	92	20 (1–58)	PFS	Univariate	SC
Takayama K^12^	2014	Japan	Prostate cancer	FFPE	IHC	Nucleus and cytoplasm	103	NR	CSS	Univariate	SC
He M^14^	2014	China	DLBCL and MALT lymphoma	FFPE	IHC	Nucleus	122	63 (3–123)	OS	Multivariate	Reported
Wong KK^28^	2014	UK	DLBCL	TMA	IHC	Nucleus	157	NR	OS	Multivariate	Reported
Wong KK^28^	2014	UK	DLBCL	TMA	IHC	Nucleus	157	NR	PFS	Multivariate	Reported
Tzankov A^29^	2015	Switzerland	DLBCL	FFPE	IHC	Nuclear	116	53 (NR)	OS	Multivariate	Reported
De Smedt L^36^	2015	Belgium	Colorectal cancer	FFPE	IHC	Nucleus and cytoplasm	165	NR	OS	Univariate	SC
Hu Z^37^	2015	China	Epithelial ovarian cancer	FFPE	IHC	Nucleus	92	NR (41–90)	OS	Multivariate	Reported
De Smedt L^36^	2015	Belgium	Colorectal cancer	FFPE	IHC	Nucleus and cytoplasm	165	NR	PFS	Univariate	SC

UK: United Kingdom; DLBCL: Diffuse large B-cell lymphoma; MALT: Mucosa-associated lymphoid tissue; FFPE: Formalin fixed paraffin-embedded; TMA: Tissue microarray; IHC: Immunohistochemistry; NR: Not reported; OS: Overall survival; RFS: Relapse-free survival; PFS: Progress-free survival; DFS: Disease-free survival; CSS: Cancer-specific survival; FFS: Failure-free survival; SC: Survival curve; HR: Hazard ratio.

**Table 2:** The cut-off values for decreased FOXP1 protein expression.

First author	Cancer type	Cut-off value
Barrans SL^15^	DLBCL	Negative or weak expression in a variable proportion of tumor cells
Fox SB^30^	Breast cancer	Negative or weak staining in neoplastic cell nuclei
Banham AH^24^	DLBCL	<30% of the cells are positive
Sagaert X^25^	DLBCL	Occasional cells have weak nuclear expression
Giatromanolaki A^17^	Endometrial cancer	<10% of cancer cells have nuclear FOXP1 expression / <50% of cancer cells have cytoplasmic FOXP1 expression
Nyman H^20^	DLBCL	Not all of the cells have strong and uniform nuclear expression
Han SL^32^	MALT lymphoma	Occasional cells have weak nuclear expression
Rayoo M^16^	Breast cancer	Negative or weak staining in the nucleus
Hoeller S^26^	DLBCL	<47.5% immunopositive tumor cells
Zhai L^33^	MALT lymphoma	<=25% of the tumor cells stain positive
Yu B^13^	DLBCL	Occasional cells with weak nuclear expression
Jiang W^34^	MALT lymphoma	<30% of the cells are positive
Zhang Y^35^	Hepatocellular carcinoma	Staining scores of 0 to 2
Feng J^18^	NSCLC	Staining score of 0 to 2
Ijichi N^31^	Breast Cancer	Immunoreactivity scores of 0 or 2
Hu CR^27^	DLBCL	<=30% of the tumor cells have nuclear staining
Takayama K^12^	Prostate cancer	Labeling index < = 10
He M^14^	DLBCL and MALT lymphoma	<=10% positive cells
Wong KK^28^	DLBCL	<70% positivity in the nuclei of tumor cells
Tzankov A^29^	DLBCL	<50% of tumor cells are positive for expression
Hu Z^37^	Epithelial ovarian cancer	Negative or weak/focal staining in nuclei
De Smedt L^36^	Colorectal cancer	All tumor cells tested negative for FOXP1expression

DLBCL: Diffuse large B-cell lymphoma; MALT: Mucosa-associated lymphoid tissue.

**Table 3:** Meta-analysis the results regarding the association between decreased expression of FOXP1 protein and OS in all tumor patients included in this study (random-effects model for meta-analyses).

Categories	Subgroups	Number of datasets	HR (95% CI)	p-Value	Heterogeneity	
					I^2^	p-Value
All		20	0.75 (0.48–1.17)	0.203	84.1%	<0.001
Year	Before 2000	8	0.91 (0.49–1.68)	0.761	79.5%	<0.001
	After 2000	12	0.65 (0.34–1.24)	0.191	87.0%	<0.001
Patient source	Asia	10	0.62 (0.29–1.30)	0.206	88.7%	<0.001
	Europe	8	0.96 (0.55–1.67)	0.891	67.6%	0.003
	North America	1	0.29 (0.15–0.55)	<0.001	—	—
	Oceania	1	1.75 (1.01–3.03)	0.046	—	—
Cancer type	**Lymphomas**	**11**	**0.37 (0.28**–**0.49)**	**<0.001**	**23.7%**	**0.218**
	**Solid tumors**	**9**	**1.82 (1.18**–**2.83)**	**0.007**	**67.3%**	**0.002**
Sample source	FFPE	14	0.67 (0.39–1.16)	0.156	79.0%	<0.001
	TMA	6	0.93 (0.44–1.97)	0.853	90.0%	<0.001
Expression location	**Nucleus**	**14**	**0.53 (0.32**–**0.86)**	**0.011**	**80.3%**	**<0.001**
	Nucleus and cytoplasm	5	1.60 (0.76–3.40)	0.218	81.8%	<0.001
	Cytoplasm	1	2.12 (0.74–6.04)	0.160	—	—
Sample size	More than 100	12	0.87 (0.52–1.45)	0.592	83.7%	<0.001
	Less than 100	8	0.59 (0.25–1.42)	0.240	85.4%	<0.001
Analysis method	Univariate	10	0.57 (0.32–1.01)	0.053	71.9%	<0.001
	Multivariate	10	0.96 (0.53–1.75)	0.891	87.2%	<0.001

FFPE: Formalin fixed paraffin-embedded; TMA: Tissue microarray; HR: Hazard ratio; CI: Confidence intervals.

**Table 4:** Meta-analysis results of the association between decreased FOXP1 protein expression and OS in patients with lymphomas.

Categories	Subgroups	Number of datasets	HR (95% CI)	p-Value	Heterogeneity	
					I^2^	p-Value
All^F^		11	0.38 (0.30–0.48)	<0.001	23.7%	0.218
Year^F^	Before 2000	4	0.41 (0.28–0.61)	<0.001	25.0%	0.261
	After 2000	7	0.36 (0.26–0.49)	<0.001	31.8%	0.185
Patient source^F^	Asia	6	0.32 (0.23–0.46)	<0.001	23.7%	0.256
	Europe	4	0.51 (0.34–0.76)	0.001	0.0%	0.393
	North America	1	0.29 (0.15–0.55)	<0.001	—	—
Cancer type^R^	DLBCL	7	0.39 (0.29–0.54)	<0.001	10.5%	0.349
	**MALT lymphoma**	**3**	**0.26 (0.11–0.59)**	**0.001**	**53.0%**	**0.119**
	DLBCL and MALT lymphoma	1	0.51 (0.25–1.04)	0.064	—	—
Sample source^F^	FFPE	9	0.37 (0.28–0.50)	<0.001	30.8%	0.172
	TMA	2	0.39 (0.25–0.61)	<0.001	34.7%	0.216
Expression location^F^	Nucleus	11	0.38 (0.30–0.48)	<0.001	23.7%	0.218
Sample size^F^	More than 100	6	0.45 (0.33–0.61)	<0.001	5.4%	0.382
	Less than 100	5	0.28 (0.19–0.42)	<0.001	10.4%	0.347
Analysis method^F^	Univariate	7	0.36 (0.26–0.50)	<0.001	39.0%	0.132
	Multivariate	4	0.40 (0.28–0.57)	<0.001	4.4%	0.371

^F^ For fixed-effects model; ^R^ For random-effects model; DLBCL: Diffuse large B-cell lymphoma; MALT: Mucosa-associated lymphoid tissue; FFPE: Formalin fixed paraffin-embedded; TMA: Tissue microarray; HR: Hazard ratio; CI: Confidence intervals.

**Table 5:** Meta-analysis results of association between decreased FOXP1 protein expression and OS in patients with solid tumors.

Categories	Subgroups	Number of datasets	HR (95% CI)	p-Value	Heterogeneity	
					I^2^	p-Value
All^R^		9	1.82 (1.18–2.83)	0.007	67.3%	0.002
Year^R^	Before 2000	4	1.77 (1.24–2.51)	0.002	0.0%	0.929
	After 2000	5	1.81 (0.80–4.13)	0.155	83.3%	<0.001
Patient source^R^	Asia	4	1.81 (0.67–4.89)	0.241	87.5%	<0.001
	Europe	4	1.79 (1.18–2.72)	0.006	0.0%	0.928
	Oceania	1	1.75 (1.01–3.03)	0.046	—	—
Cancer type^F^	Breast cancer	3	1.76 (1.22–2.55)	0.003	0.0%	0.690
	Endometrial cancer	2	2.24 (0.97–5.21)	0.060	0.0%	0.858
	Hepatocellular carcinoma	1	0.46 (0.24–0.88)	0.018	—	—
	**NSCLC**	**1**	**3.11 (1.87**–**5.17)**	**<0.001**	—	—
	Colorectal cancer	1	1.86 (0.67–5.19)	0.235	—	—
	Epithelial ovarian cancer	1	2.81 (1.44–5.47)	0.002	—	—
Sample source^R^	FFPE	5	2.47 (1.60–3.82)	<0.001	0.0%	0.964
	TMA	4	1.44 (0.69–3.03)	0.332	85.8%	<0.001
Expression location^R^	Nucleus	3	2.15 (1.43–3.22)	<0.001	0.0%	0.549
	Nucleus and cytoplasm	5	1.60 (0.76–3.40)	0.218	81.8%	0.000
	Cytoplasm	1	2.12 (0.74–6.04)	0.160	—	—
Sample size^R^	More than 100	6	1.62 (0.91–2.90)	0.105	77.3%	0.001
	Less than 100	3	2.58 (1.53–4.35)	<0.001	0.0%	0.905
Analysis method^R^	Univariate	3	2.08 (1.09–3.99)	0.027	0.0%	0.947
	Multivariate	6	1.75 (0.99–3.10)	0.057	79.3%	<0.001

^F^ For fixed-effects model; ^R^ For random-effects model; NSCLC: Non-small cell lung cancer; FFPE: Formalin fixed paraffin-embedded; TMA: Tissue microarray; HR: Hazard ratio; CI: Confidence intervals.
