# Supplementary material for: Prognostic value of decreased FOXP1 protein expression in various tumors: a systematic review and meta-analysis
Source: Sci Rep. 2016 Jul 26;6:30437. doi: 10.1038/srep30437 (PMC4960649; doi:10.1038/srep30437)
Supplement: Supplementary Information [file srep30437-s1.pdf]

Prognostic value of decreased FOXP1 protein expression in various tumors: a systematic review and meta-analysis

Jian Xiao[1], Bixiu He[1], Yong Zou[1], Xi Chen[2], Xiaoxiao Lu[1], Mingxuan Xie[1], Wei Li[1], Shuya He[3], Shaojin You[4], Qiong Chen[1,]

[1]Department of Geriatrics, Respiratory Medicine, Xiangya Hospital of Central South University, Changsha, China
[2]Department of Respiratory Medicine, Xiangya Hospital of Central South University, Changsha, China
[3]Departmentof Biochemistry and Biology, University of South China, Hengyang, China
[4]Laboratory of Cancer Experimental Therapy, Atlanta Research & Educational Foundation (151F), Atlanta VA Medical Center, Decatur, GA, USA

Correspondence and requests for materials should be addressed to Q. C. (email: qiongch@163.com)

Supplementary Table S1. Meta-regression analysis results of OS.

Categories	Residual I^2	Adjusted R^2
Publication year	84.93%	-7.47%
Country	87.20%	-2.73%
Cancer type	**6.26%**	**100.00%**
Sample source	83.94%	-3.77%
Expression location	**80.68%**	**24.29%**
Sample size	83.82%	6.47%
Analysis method	82.45%	4.36%
